# A novel TLR2-triggered signalling crosstalk synergistically intensifies TNF-mediated IL-6 induction

**DOI:** 10.1111/jcmm.12294

**Published:** 2014-04-24

**Authors:** Yu-Ling Chang, Tzu-Hui Chen, Yi-Hsiu Wu, Guann-An Chen, Tzu-Huei Weng, Ping-Hui Tseng, Shie-Liang Hsieh, Shu-Ling Fu, Chi-Hung Lin, Chun-Jen Chen, Ching-Liang Chu, Iok In Christine Chio, Tak Wah Mak, Nien-Jung Chen

**Affiliations:** a Institute of Microbiology and Immunology, School of Life Sciences, National Yang-Ming UniversityTaipei, Taiwan; b Institute of Biochemistry and Molecular Biology, School of Life Sciences, National Yang-Ming UniversityTaipei, Taiwan; c Inflammation and Immunity Research Center, National Yang-Ming UniversityTaipei, Taiwan; d Institute of Clinical Medicine, School of Medicine, National Yang-Ming UniversityTaipei, Taiwan; e Genomics Research Center, Academia SinicaTaipei, Taiwan; f Institute for Cancer Biology and Drug Discovery, College of Medical Science and Technology, Taipei Medical University and Immunology Center, Taipei Veterans General HospitalTaipei, Taiwan; g Institute of Traditional Medicine, School of Medicine, National Yang-Ming UniversityTaipei, Taiwan; h Department of Biochemical Science and Technology, College of Life Science, National Taiwan UniversityTaipei, Taiwan; i Graduate Institute of Immunology, College of Medicine, National Taiwan UniversityTaipei, Taiwan; j The Campbell Family Institute for Breast Cancer Research, Ontario Cancer Institute, University Health Network and Department of Medical Biophysics, University of TorontoToronto, ON, Canada

**Keywords:** Toll-like receptor, tumour necrosis factor, signalling crosstalk, TRAF6, TRADD

## Abstract

Toll-like receptors (TLR) recognize pathogens and trigger the production of vigorous pro-inflammatory cytokines [such as tumour necrosis factor (TNF)] that induce systemic damages associated with sepsis and chronic inflammation. Cooperation between signals of TLR and TNF receptor has been demonstrated through the participation of TNF receptor 1 (TNFR) adaptors in endotoxin tolerance. Here, we identify a TLR2-mediated synergy, through a MyD88-independent crosstalk, which enhances subsequent TNF-mediated nuclear factor-kappa B activation and interleukin-6 induction. Membrane-associated adaptor MAL conduces the link between TNF receptor-associated factor 6 (TRAF6) and TNFR-associated death domain, leading to a distinctive K63-ubiquitinylated TRAF6 recruitment into TNFR complex. In summary, our results reveal a novel route of TLR signal that synergistically amplifies TNF-mediated responses, indicating an innovative target for inflammation manipulation.

## Introduction

Microbial infection-triggered inflammation has been linked with various autoimmune and inflammatory diseases. Toll-like receptors sense pathogens and mediate the activation of nuclear factor kappa-light-chain-enhancer of activated B cells (NF-κB), interferon-regulatory factor (IRF), and mitogen-activated protein kinase (MAPK) signals [1]. Oppositely, excessive TLR activation accompanied with superfluous inflammatory cytokines may result in undesirable tissue damages, leading to the inflammatory diseases [2]. Tightly regulated TLR signals in cooperation with downstream effectors are required to properly control the mode and intensity of innate immunity [3].

Both myeloid differentiation primary-response protein 88 (MyD88)-dependent and TIR-domain-containing adaptor protein inducing interferon (IFN)-β (TRIF)-dependent pathways have been identified in TLR signalling [4]. Most TLRs (except TLR3) associate MyD88 to recruit members of interleukin-1 receptor-associated kinase family, followed by the recruitment of TRAF6, resulting in MAPK and NF-κB activation [1,5]. Alternatively, TRIF-mediated signal was found downstream of TLR3 and TLR4, leading to the activation of IRF3 and type I interferon production [4]. Distinctively, TLR4 recruits additional adaptors and triggers both MyD88 and TRIF signals. A membrane-associated adaptor MyD88-adaptor-like protein (MAL) with N-terminal phosphatidylinositol 4,5-bisphosphate (PIP_2_)-interacting domain was identified upstream of MyD88-pathway upon TLR4 and TLR2 activation [6]. Phosphorylated MAL was also found to directly interact with TRAF6 to activate NF-κB [7]. Other than MAL, TRIF-related adaptor molecule (TRAM) was also found in TLR4 signalling upstream of TRIF route [8]. Evidence gained from dynasore™-treated cells suggested that TLR4 sequentially activates MAL-MyD88 and TRAM-TRIF pathways through a compartmental regulation: MAL-MyD88 complex at the cell membrane [9] *versus* TRAM-TRIF complex at the endosome [10].

Tumour necrosis factor, a pleiotropic pro-inflammatory cytokine which is acutely generated by innate cells upon TLR triggering [11], activates inflammation mainly through TNFR1. Beneficially, TNF activates endothelial cells and promotes leucocyte infiltration leading to local inflammation. However, excessive TNF may harmfully induce systemic host-attack causing diseases [12]. TLR-triggered septic mortality is significantly decreased in mice deficient for TNF or TNFR1 in comparison with wild-type (WT) mice, indicating that TNF-TNFR1 signals mediate the deleterious effects of endotoxin toxicity [12]. Signalling wise, TNF can trigger NF-κB and MAPK activation to regulate cell proliferation and survival. Oppositely, it may mediate cell apoptosis through inducing death-inducing signalling complex (DISC) formation [12,13]. Upon stimulation, TNFR-associated death domain (TRADD) interacts with TNFR1 [14], further recruits TRAF2 and receptor-interacting protein 1 (RIP1) to mediate the NF-κB activation [15]. Subsequently after receptor internalization, a second protein complex [containing liberated TRADD and Fas-associated *via* death domain (FADD)] is formed which activates caspase-8 resulting in cell apoptosis [13,16].

In reality during infection, multiple pathogen recognition receptors are simultaneously triggered by various microbial ligands. Signalling crosstalk sequentially triggered by TLR4 and TLR9 amplifies the activation of macrophages [17]. Besides, precursory IL-1 receptor signalling was found beneficial for facilitating TLR9-mediated type I interferon production [18]. Paradoxically, prolonged treatment of TLR agonist induces ‘TLR tolerance’ both *in vitro* [19] and *in vivo* [20] *via* the negative regulators such as inhibitor of κB (IκB) and A20 [21,22]. Furthermore, cooperation of TLR and its downstream effectors can also crucially regulate innate immunity [3]. A secondary signal, such as IFN-γ or TLR stimulation, is required to trigger the optimal TNF-mediated responses [23–25]. However, the details of involved mechanism mostly remain to be investigated.

The synergistic cooperation between signals of TLR and TNFR were mainly found through the participation of TNFR1-downstream adaptors (RIP1, FADD and TRAFs) in TLR signalling [26–28]. We and other groups previously found that TRADD can play a crucial role in TRIF-mediated NF-κB activation downstream of TLR3 and TLR4 [29–31]. Intriguingly, interactions between TRADD with TLR4-TIR and MAL were observed [29], implying an un-clarified function of TRADD proximally to the membrane compartment of TLR signalsome. Recently, a communication from TNFR to TLR signalling was demonstrated for eliciting TLR tolerance *via* GSK3 activation which leads to chromatin re-modification and transcriptional up-regulation of NF-κB inhibitors [32]. In the present study, we investigate the crosstalk between TLR and TNF signals, and report a novel link *via* cytosolic adaptors which contributes to the optimal IL-6 induction mediated by sequential TLR2 and TNF stimulations.

## Materials and methods

### Reagents

Recombinant murine IL-1β and TNF were obtained from PeproTech (Rocky Hill, NJ, USA). Recombinant mouse macrophage colony-stimulating factor (M-CSF) was purchased from R&D Systems® (Minneapolis, MN, USA). Lipopolysaccharide (LPS), Pam3Csk4, and Poly(I:C) were purchased from InvivoGen (San Diego, CA, USA). Antibodies to c-Myc (9E10), TRADD (H278), ERK2 (D2) and β-tubulin (H235) were obtained from Santa Cruz Biotechnology®, Inc (Dallas, TX, USA). Antibodies specific for phospho-JNK/SAPK (Thr183/Tyr185), phospho-p44/42 MAPK (pERK) and phospho-IκB (Ser32/36) (5A5) were purchased from Cell Signalling Technology® (Danvers, MA, USA). Antibodies to Na^+^-K^+^ ATPase (ab69312) and green fluorescent protein (GFP; ab7671) were purchased from Abcam® (Cambridge, UK). Antibody to GAPDH was obtained from Sigma-Aldrich® (St. Louis, MO, USA).

### Mice

Wild-type C57BL/6 mice, *Tradd*, *Tnf* and *Myd88* knockout mice have been described previously [29,33,34] and were maintained in the laboratory animal centre at NYMU following the instruction of Animal Care and Use Committee. For mouse typing, genomic DNA samples were prepared from tail tissues harvested from mice and genotyped by PCR by using specific primer sets.

### Primary bone marrow-derived macrophage preparation

Bone marrow cells harvested from tibia and femur bones were collected. After removing red blood cells with a hypotonic ACK buffer (0.155 M NH_4_Cl, 10 mM KHCO_3_ and 0.1 mM EDTA, pH 8.0; final pH was adjusted to 7.4 and then sterilized with 0.22 μm filter), the mononuclear cells were carefully harvested, washed, counted and then cultured with 6 ml RPMI containing M-CSF (20 ng/ml) and 10% fetal bovine serum (FBS) at Petri dishes (9-cm) for macrophage differentiation (5 × 10^6^ cells/dish). Fresh medium (3 ml) with M-CSF were added into original culture every other day. On day 5, the attached bone marrow-derived macrophages (BMDMs) were collected with 10 ml ice-cold PBS contains 2 mM EDTA and then re-plated for stimulation.

### Cell culture

HEK293T cells were cultured in DMEM with 5% FBS at 10 cm cell culture dishes. Embryonic fibroblasts (MEFs) were derived from mouse embryos as previous described [29] and cultivated in DMEM with 10% FBS at 10 cm cell culture dishes at a 37°C incubator with 5% CO_2_. One million cells were initially seeded, cultured for 3 days and detached by trypsin-EDTA, counted by trypan-blue exclusion method on Countess™ cell counter (Thermo Fisher Scientific, Waltham, MA, USA) and seeded into new plates for further experiments.

### Virus production and gene knockdown

To express TRADD in primary cells, TRADD cDNA was isolated and subcloned into pBabe.puro vector for retrovirus production. The vectors were transfected into Phoenix lines, the culture media containing recombinant virus were harvested after 24–48 hrs and were used for primary cell infection following the protocols as previous described [35]. The lentivirus-based specific gene knockdown constructs were obtained from National RNAi Core Facility located at the Institute of Molecular Biology/Genomic Research Center, Academia Sinica. The lentiviral production and infection were following the protocols provides by vendor (http://rnai.genmed.sinica.edu.tw/webContent/web/protocols). In briefly, shRNA in pLKO.1 vector and helper vectors were transfected into HEK293T cells, and the recombinant lentivirus-containing media were harvested at 40 and 64 hrs after transfection. Titrated virus-containing media were used for MEF infection. Puromycin were added into MEF culture 24 hrs after infection to remove the non-infected cells.

### Protein lysate preparation

Cells were washed with PBS and then harvested by scraping method into centrifuge tubes. Cell pellets were lysed with RIPA (50 mM Tris-HCl pH 8.0, 150 mM NaCl, 1% NP-40, 0.5% deoxycholate, 0.1% SDS) or IP buffer (20 mM Tris-HCl pH8.0, 150 mM NaCl, 0.2% NP-40, 10% glycerol) with protease inhibitor cocktail (Roche Diagnostics Corporation, Indianapolis, IN, USA) at 4°C for 30 min. After thoroughly mixed of lysate, the samples were spun at 20,000 × g, 10 min. and 4°C on a bench-top centrifuge. Finally, supernatants were collected into new tubes and quantified by bicinchoninic acid Protein Assay Reagent (Thermo Fisher Scientific).

### Subcellular fractionation

The membrane fractionation was prepared as described [36]. MEFs (2 × 10^6^/plate) were cultured in a 10-cm tissue culture dish. After indicative treatment or transfection, cells were washed with ice-cold PBS. Appropriate amount of subcellular fractionation buffer (20 mM HEPES pH 7.4, 10 mM KCl, 1.5 mM MgCl_2_, 1 mM EDTA, 1 mM EGTA with protease inhibitors) was added. Cells were scrapped from dishes and collected into 1.5 ml Eppendorf tubes. After spun down, cells were broken by 30G syringes. The unbroken cells and nuclear pellet were then separated by centrifugation at 500 × g for 15 min. at 4°C. Supernatants were collected into new tubes and further centrifuged at 20,000 × g for 30 min. at 4°C. The supernatants were collected as cytosol fraction and the pellets were membrane part which were further collected and re-suspended in RIPA buffer.

### Immunoprecipitation

Adequate amounts of immunoprecipitate antibody were pre-incubated with protein G beads (1 μg Ab/20 μl beads/sample) with gentle rotation at 4°C for 1 hr. The aliquot of Ab-beads mixture was prepared into microcentrifuge tubes with quantified equal amount of total cell lysates (1 mg of total proteins in IP buffer) and further incubated at 4°C with rotation for overnight. To wash away unbound protein, the samples were centrifuged at 800 × g for 5 min. to remove the supernatant, and the beads were re-suspended with 1 ml of ice-cold IP buffer then transferred into new tubes. Beads-bound Ab-associated targeted protein complex was then analysed by SDS-PAGE and Western blotting after five times of wash and transfer step.

### SDS-PAGE and Western blotting

Samples containing equal amounts of protein were fractionated on a 10% SDS-PAGE gel and transferred onto a Hybond™-P membrane (GE Healthcare, Little Chalfont, UK) by using Trans-Blot cell (Bio-Rad Laboratories, Hercules, CA, USA). The membrane was then blocked with blocking solution (5% skim milk or 1% BSA in TBS-T as vendor's suggestion) at room temperature for 1 hr followed by the incubation with titrated primary-antibody-containing blocking solution at 4°C overnight. On the second day, the blot was washed three times with 10 ml TBS-T for 10 min., and then incubated with titrated HRP-conjugated secondary antibody (obtained from GE Healthcare and Santa Cruz Biotechnology®, Inc.) in blocking solution (5% skim milk in TBS-T) for at least 1 hr. After TBS-T washes (also 10 min., three times), the target protein signals on the membrane were visualized by chemiluminescence reagent (Merck Millipore, Billerica, MA, USA) exposed on X-ray films (MidSci, St. Louis, MO, USA). For image signal quantification, the scanned gel TIFF files were further analysed by using the ImageJ software.

### NF-κB reporter assay

HEK293T cells or MEF cells were seeded on 24-well dish (10^5^ cells/well), and then co-transfected with desired gene, NF-κB reporter pGL4.32 (0.1 μg/well), and CMV-β-galactosidase (0.1 μg/well) by calcium phosphate precipitation method. Twenty-four to 48 hrs after transfection, cells were washed with ice-cold PBS and lysed within lysis buffer provided by Promega Luciferase Reporter System. After freezing and thawing, the samples were harvested and centrifuged to collect the cell lysate from supernatant. The mixture of 20 μl lysate and 50 μl of luciferase substrate were used for luciferase activity analysis. Transfection efficiency was normalized by β-galactosidase activity determined by ONPG assay.

### Cytokine ELISA

The culture supernatants were collected at 24 hrs after stimulation and stored at −80°C. IL-6 concentration in the harvested samples was determined by IL-6 ELISA set (BD Bioscience, San Jose, CA, USA) according to vendor's instruction.

### RNA purification and RT-PCR

Cells were rinsed with PBS and lysed with Trizol reagent (Thermo Fisher Scientific). Total RNA was purified according to vendor's instructions and cDNA was prepared by High Capacity cDNA Reverse Transcription Kits (Thermo Fisher Scientific) as the template in PCR reaction performed by using DreamTaq™ System (Thermo Fisher Scientific). Finally, amplifying signals were checked by agarose gel electrophoresis.

### Statistical analysis

Data were expressed as mean ± SD. The significance of difference in mean between different treatment groups were tested by one-way (for two parameters) or two-way anova (for multiple parameters; as indicative in legends) with scheffe method for post hoc analysis using IBM® SPSS® statistics Version 20 software (IBM, Armonk, NY, USA). The valued *P* < 0.05 were considered statistically significant. The statistical significance was presented as: *P < 0.05; **P < 0.01; ***P < 0.001.

## Results

### TLR triggering enhances subsequent TNF-mediated NF-κB activation and IL-6 production in macrophages and fibroblasts

**Fig. 1:**
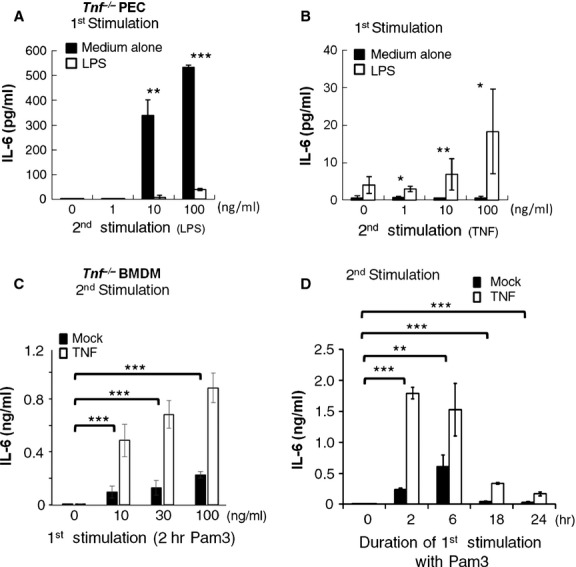
Pre-treatment of Pam3Csk4 boosts subsequent tumour necrosis factor (TNF)-mediated interleukin (IL)-6 production in macrophages. (**A** and **B**) *Tnf*^−/−^ peritoneal macrophages (PEC) were pre-stimulated for 24 hrs with 100 ng/ml of lipopolysaccharide (LPS) and subsequently stimulated for 24 hrs with indicative doses of LPS (**A**) for ‘LPS tolerance’ and TNF (**B**) for toll-like receptor-TNF receptor (TLR-TNFR) signalling cross-talk examination. The result is shown as mean ± SD; *n* = 3. The statistical significances were analysed by One-way anova. (**C** and **D**) *Tnf*^−/−^ bone marrow-derived macrophages (BMDM) pre-treated with (**C**) various doses of Pam3Csk4 for 2 hrs or (**D**) 30 ng/ml of Pam3Csk4 for indicative duration, and then stimulated with 10 ng/ml of TNF for 24 hrs. The TNF-mediated IL-6 production was determined by ELISA. (**C** and **D**) The result is shown as mean ± SD; *n* = 3 per experimental condition. The statistical significances of different groups between Pam3Csk4 pre-treatment and none treatment were analysed by two-way anova.

It was recently reported that the communication from TNFR to TLR signalling is helpful to elicit TLR tolerance [32], leading us to examine the role of endogenous TNF in the LPS-mediated TLR tolerance by using cells with TNF deficiency. Peritoneal macrophages (PEC) from *Tnf* deficient (*Tnf*^−/−^) mice were first primed with or without LPS for 24 hrs and then stimulated with serial amounts of LPS for 24 hrs, and the IL-6 induction were determined. Our result showed that TLR tolerance is still existed in *Tnf*^−/−^ macrophages (Fig. 1A), suggesting that without TNF, other inhibitory factors still could mediate the TLR tolerance. In parallel, we also examined the effects of LPS pre-treatment on subsequent TNF-induced IL-6 production by adding back recombinant murine TNF in the culture of LPS-primed *Tnf*^−/−^ macrophages. Intriguingly, a drastic enhancement on TNF-mediated IL-6 induction is induced in cells receiving LPS pre-treatment compared to those without pre-treatment (Fig. 1B), implicating a signalling synergy is triggered by LPS to intensify the sequential TNF-mediated responses as previously suggested [23–25].

Lipopolysaccharide triggers TLR4-mediated signalling through both MyD88- and TRIF-dependent pathways. Pro-inflammatory cytokine induction is mainly regulated by the MAL-MyD88 route which can also be triggered by TLR2 without turning on the TRIF-arm. Therefore, we further used Pam3Csk4 (a TLR1/TLR2 agonist) as the precursory stimulus to activate BMDMs. Accordingly, pre-treatment with Pam3Csk4 resulted in an augmentation of sequential TNF-induced IL-6 production in both WT (data not shown) and *Tnf*^−/−^ BMDMs in a dose-dependent manner (Fig. 1C). To be noticed, 30 min. of TLR2 stimulation is sufficient to facilitate TNF-mediated IL-6 production (data not shown), and 2 hrs TLR2 pre-stimulation induces the maximum boost (Fig. 1D).

**Fig. 2:**
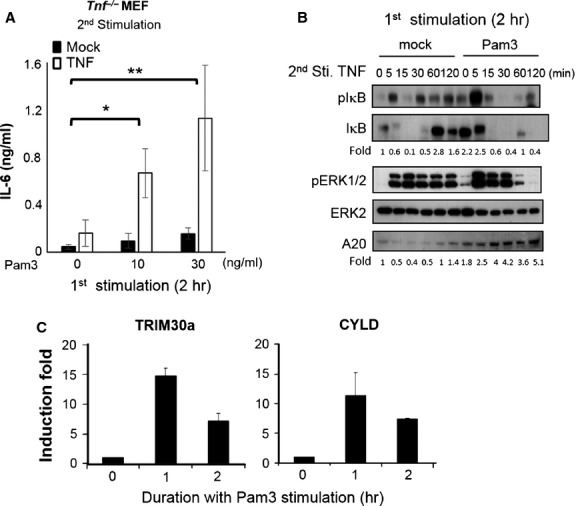
Pre-treatment of Pam3Csk4 boosts tumour necrosis factor (TNF)-mediated interleukin (IL)-6 production and nuclear factor-kappa B (NF-κB) activation in *Tnf*^−/−^ MEFs. *Tnf*^−/−^ MEFs were pre-treated with (**A**) various doses of Pam3Csk4 for 2 hrs, washed and then stimulated with 10 ng/ml of TNF for 24 hrs. The TNF-mediated IL-6 production was determined by ELISA in the supernatants. The result is shown as mean ± SD; *n* = 4. The statistical significances of different groups between Pam3Csk4 pre-treatment and control were analysed by two-way anova. (**B**) *Tnf*^−/−^ MEFs were pre-treated with 30 ng/ml of Pam3Csk4 for 2 hrs, washed and then stimulated with 10 ng/ml of TNF for indicative time. Activation of NF-κB (phosphorylated IκB) and ERK (phosphorylated ERK1/2), as well as the levels of IκB and A20 were determined by Western blotting and quantified by using ImageJ software normalized to total ERK2 signal (internal loading controls). A representative of three independent experiments is shown. (**C**) The mRNA levels of inhibitors TRIM30a and CYLD in MEFs treated with Pam3Csk4 were determined by qRT-PCR (representative from two independent experiments).

A similar synergy on TNF-mediated IL-6 induction can also be observed in TLR2 pre-stimulated *Tnf*^−/−^ MEFs (Fig. 2A). With 2 hrs of Pam3Csk4 pre-treatment, the basal phosphorylation of IκB and ERK1/2 are slightly enhanced. By contrast, the total amount of IκB is significantly increased in pre-treated MEFs (Pam3 0 min. compared to mock 0 min., Fig. 2B), while the total level of ERK2 remains unchanged. Stimulation of TNF triggers more NF-κB activation in Pam3Csk4 pre-treated MEFs (with higher levels of phospho-IκB at 5 and 15 min. of stimulation, and also prolonged degradation of IκB until 1–2 hrs). In addition, an early enhancement of ERK activation is also observed (Fig. 2B). NF-κB signals mediated by TLR and TNFR have been found negatively regulated by inhibitors, such as IκB [21], A20 [22], tripartite motif-containing 30A (TRIM30a) [37] and deubiquitinases cylindromatosis protein (CYLD) [38]. However, the protein levels of IκB and A20 (Fig. 2B) as well as the mRNA levels of TRIM30a and CYLD (Fig. 2C) are significantly up-regulated, rather than down-regulated, in TLR2-stimulated MEFs, suggesting that the synergy we observed is not a consequence of down-regulating the NF-κB inhibitors.

### MAL, but not MyD88, is crucial for TLR2-mediated synergy

**Fig. 3:**
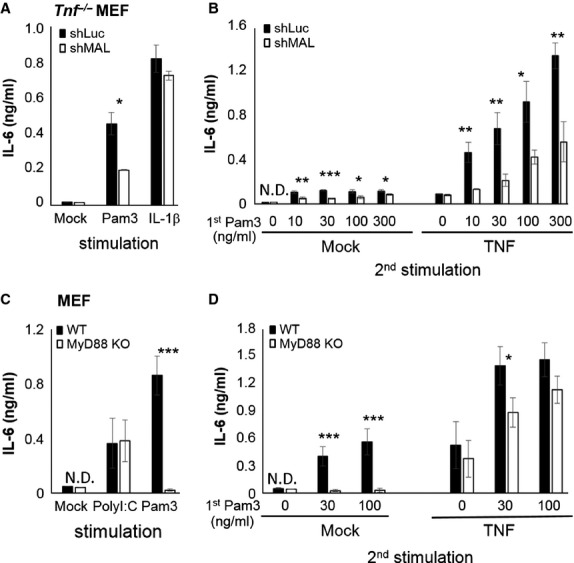
MAL, but not MyD88, is crucial for TLR2-mediated synergy on tumour necrosis factor (TNF) responses. (**A** and **B**) Stably *Mal* knockdown *Tnf*^−/−^ MEF clones were established by lentiviral delivered shMAL (or control shRNA for luciferase; shLuc). (**A**) The efficacy of *Mal* knockdown is confirmed by determining the induction of interleukin (IL)-6 stimulated by TNF (10 ng/ml), Pam3Csk4 (30 ng/ml) and IL-1β (10 ng/ml). (**B**) The selected clones were pre-treated with indicative doses of Pam3Csk4 for 2 hrs, washed and subsequently stimulated with TNF (10 ng/ml) overnight. (**C**) The effect of MyD88 depletion in MEFs is shown by comparing the IL-6 induction in *Myd88*^−/−^ or WT MEFs overnight stimulated with Pam3Csk4 (30 ng/ml) and poly (I:C; 10 μg/ml). (**D**) *Myd88*^−/−^ or WT MEFs were pre-treated with indicative Pam3Csk4 for 2 hrs, washed and then stimulated with TNF (10 ng/ml) overnight. The IL-6 levels in supernatants were determined by IL-6 ELISA. The result is shown as mean ± SD and the statistical significances of groups between WT and *Mal* knockdown *Tnf*^−/−^ MEFs (**A** and **B**; *n* = 3) or *MyD88* KO MEFs (**C** and **D**; *n* = 4) were analysed by one-way anova.

To investigate the role of common signal components in the synergy process induced by TLR2 and TLR4, stably knockdown clones for *Mal* (shMAL) and scramble control luciferase knockdown (shLuc) in *Tnf*^−/−^ MEFs were established, confirmed by MAL mRNA depletion (data not shown) and also by losing response to Pam3Csk4, but remained sensitive to IL-1β and TNF stimulation (Fig. 3A and B). After sequential stimulation of Pam3Csk4 and TNF, control shLuc cells showed a significant synergy by TLR2 on TNF-mediated IL-6 induction which was drastically reduced in shMAL cells (Fig. 3B), indicating that MAL plays a crucial role in the synergic process.

The role of MyD88 in the synergy was also examined by using MEFs derived from *Myd88* knockout (*Myd88*^−/−^) mice [34]. These cells were insensitive to Pam3Csk4 stimulation, but remained responsive to Poly(I:C) (Fig. 3C) and TNF stimulation (Fig. 3D) [39]. Intriguingly, although TLR2 stimulation could not induce IL-6 (Fig. 3C) and TNF production (data not shown) from *Myd88*^−/−^ MEFs, these cells keep the ability to induce a MyD88-independent synergy which triggers comparable levels of IL-6 production in WT and *Myd88*^−/−^ MEFs sequentially stimulated with Pam3Csk4 and TNF (Fig. 3D).

### MAL recruits TRADD to proximal membrane upon TLR2 stimulation

**Fig. 4:**
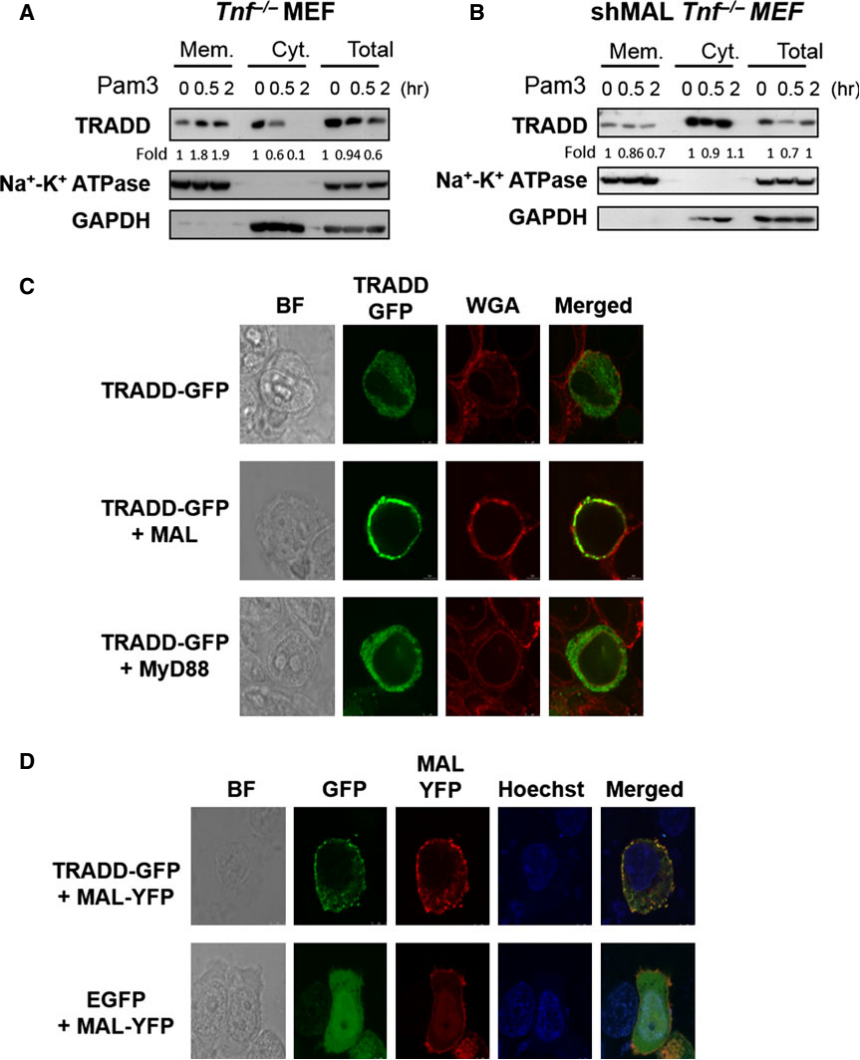
Tumour necrosis factor receptor-associated death domain (TRADD) is recruited to membrane compartment upon Pam3Csk4 stimulation through a MAL-dependent mechanism. (**A**) *Tnf*^−/−^ MEFs and (**B**) *Mal* knockdown (shMAL) *Tnf*^−/−^ MEFs were treated with 30 ng/ml of Pam3Csk4 for 0.5 or 2 hrs and then fractionated into membrane and cytosol fractions, and the levels of TRADD were determined by Western blotting. The numbers below the gel indicate the fold changes of TRADD normalized to untreated group. (**C**) HEK293T cells were cotransfected with TRADD-GFP expression vector plus control vector, vector encodes MAL or MyD88. Cells were stained by Alexa Fluor® 594 WGA at 24 hrs after transfection and then examined by confocal microscopy. (**D**) HEK293T cells were cotransfected with vectors expressing MAL-YFP plus TRADD-GFP or EGFP. The transfected cells were stained with Hoechst 33342 at 24 hrs and then examined by confocal microscopy. A representative of three independent experiments is shown.

A link between TRADD and MAL, but not MyD88, has been reported [29]. We further found that N-terminal domain of TRADD (without death domain) is capable of MAL interaction (data not shown). MAL contains an N-terminal PIP_2_-interacting motif for plasma membrane association [9], whether MAL can recruit TRADD to proximal membrane upon TLR2 stimulation was next examined by using cellular fractionation assay. The proportion of membrane-associated TRADD is gradually raised after 30 min. to 2 hrs of Pam3Csk4 treatment compared to the basal in non-stimulated cells, whereas the amount of TRADD in the whole cell lysate is gradually decreased, which is mainly contributed by the deduction of cytosolic TRADD after Pam3Csk4 stimulation (Fig. 4A). By contrast, the TLR2-induced TRADD membrane recruitment is vanished in shMAL *Tnf*^−/−^ MEFs (Fig. 4B).

The translocation of TRADD was further examined in HEK293T cells with ectopic MAL or MyD88 expression. A C-terminal enhanced GFP fused recombinant TRADD (named TRADD-GFP) was established for *in situ* detecting mouse TRADD. Intracellular distribution of TRADD-GFP was examined by confocal microscopy in transfected cells co-stained with Alexa Fluor® 594 WGA as a membrane indicator. Most of TRADD-GFP are located within cytosol in a form of minute spotted aggregates (Fig. 4C). Overexpression of MAL and MyD88, as previously reported, are capable of triggering NF-κB activation (data not shown). However, TRADD membrane-accumulation can only be observed in TRADD transfected cells co-expressing MAL, but not MyD88 (Fig. 4C). The co-localization of MAL and TRADD proximally to plasma membrane is further confirmed in cells co-expressing C-terminal fused MAL-yellow fluorescent protein (YFP) and TRADD-GFP (Fig. 4D), but is not observed in cells co-expressing MAL-YFP and control EGFP.

### Membrane recruiting of TRADD augments TNF responses

**Fig. 5:**
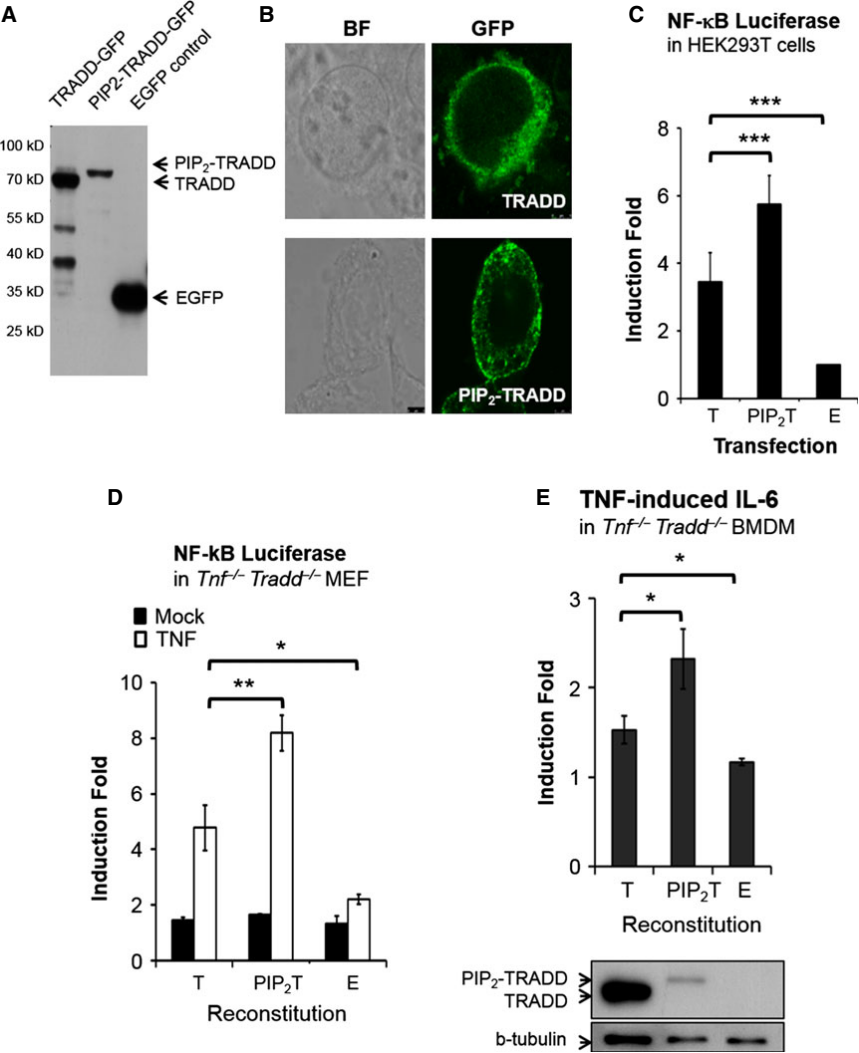
Membrane-associated PIP_2_-TRADD-GFP triggers stronger nuclear factor-kappa B (NF-κB) activation than WT TRADD-GFP does. The HEK293T cells were transfected with vectors encode WT TRADD-GFP and PIP_2_-TRADD for 24 hrs. The expression and distribution of indicative fusion proteins were examined by (**A**) Western blotting and (**B**) confocal microscopy, respectively. (C) HEK293T cells or (**D**) *Tnf*^−/−^*Tradd*^−/−^ MEF were cotransfected with NF-κB luciferase reporter and β-gal vector in the presence of TRADD-GFP or PIP_2_-TRADD-GFP expression vectors. After 24 hrs, luciferase activities were determined. Results are presented as the induction of luciferase activity relative to β-galactosidase activity. (**E**) Tumour necrosis factor (TNF)-mediated interleukin (IL)-6 induction were examined in WT TRADD-GFP- or PIP_2_-TRADD-GFP-reconstituted *Tnf*^−/−^*Tradd*^−/−^ bone marrow-derived macrophages (BMDMs). Induction fold was calculated by comparing IL-6 level produced by TNF-stimulated cells with the basal IL-6 from unstimulated cells. A representative result of two (**E**) to three (**C** and **D**) experiments is shown (mean ± SD; *n* = 3).

To determine the impact of TRADD membrane recruitment on TNF-mediated responses, a modified TRADD-GFP protein N-terminal fused with the PIP_2_-interacting motif (residues of 2-39) from MAL [9], termed PIP_2_-TRADD-GFP (previously termed cyt-TRADD) [40] was established. The expression and distribution of PIP_2_-TRADD-GFP were examined in transfected HEK293T cells by Western blotting (Fig. 5A) and confocal microscopy (Fig. 5B), respectively. To be noticed, the protein level of PIP_2_-TRADD-GFP is repeatedly found lower than WT TRADD-GFP (Fig. 5A and E), implicating an un-characterized mechanism which modulates protein stability of PIP_2_-TRADD-GFP remains to be dissected. Contrastively, a significantly higher NF-κB activity is triggered by transient expressing PIP_2_-TRADD-GFP than WT TRADD-GFP in HEK293T cells (Fig. 5C), as well as in the transiently reconstituted *Tnf*^−/−^*Tradd*^−/−^ MEFs in response to exogenous TNF stimulation (Fig. 5D). In addition, TNF-mediated IL-6 production, which is completely abolished in *Tnf*^−/−^*Tradd*^−/−^ cells, is higher under PIP_2_-TRADD-GFP reconstitution than in WT TRADD-GFP-reconstituted BMDMs (Fig. 5E). Thus, membrane-associated TRADD is capable of delivering a stronger NF-κB signal for mediating TNF-induced IL-6 production.

### TRAF6, a mediator of TLR signalling, is recruited by TRADD into TNFR1 signalsome

**Fig. 6:**
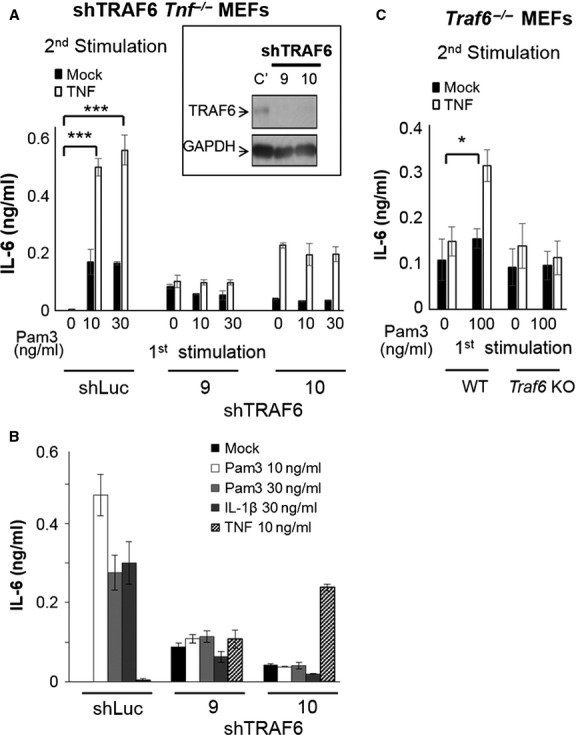
TRAF6 plays crucial roles in TLR2-mediated synergy on tumour necrosis factor (TNF)-induced interleukin (IL)-6 production. Stably *Traf6* knockdown (*shTRAF6*) *Tnf*^−/−^ MEF clones were established by lentiviral delivered shTRAF6, and the depletion of TRAF6 expression was confirmed by (**A**) Western blotting (upper box) and functionally confirmed by (**B**) overnight stimulation with Pam3Csk4 (10 and 30 ng/ml), IL-1β (30 ng/ml) or TNF (10 ng/ml). The selected (**A**) *shTRAF6 Tnf*^−/−^ MEF clones or (**C**) *Traf6*^−/−^ MEFs were pre-treated with indicative doses of Pam3Csk4 for 2 hrs, washed and subsequently stimulated with TNF (10 ng/ml) overnight. The IL-6 induction was determined by ELISA. (**A** and **C**) The result is shown as mean ± SD, *n* = 3, and the statistical significances were analysed by two-way anova.

We speculate that a TLR2-induced, MAL-TRADD-related process may modulate the composition of TNFR signalsome, leading to the synergy on TNF responses. TRAF6, a well-known positive mediator in MyD88 signalling [41] and a direct interacting partner of MAL [7], was recently found involves in TNF-mediated signalling [42]. We established the TRAF6 knockdown clones (shTRAF6) in *Tnf*^−/−^ MEFs, confirmed the knockdown efficiency by Western blotting (Fig. 6A, upper) and by monitoring the IL-6 production in response to Pam3Csk4, IL-1β (TRAF6-dependent) and TNF stimuli (Fig. 6B). As previous reports suggested, shTRAF6 *Tnf*^−/−^ MEFs do not response to Pam3CSK4 and IL-1β stimulation, but produce a slightly higher level of IL-6 in response to TNF in comparison with scramble knockdown cells (shLuc; Fig. 6B). The sequential stimulation of Pam3Csk4 and TNF, unlike in shLuc cells which showed a clear synergy, showed no enhancement on TNF-mediated IL-6 induction in shTRAF6 *Tnf*^−/−^ MEFs (Fig. 6A, lower), supporting that TRAF6 is important for TLR2-mediated synergy. The involvement of TRAF6 in the crosstalk is further demonstrated in TRAF6 knockout (*Traf6*^−/−^) MEFs [43]. In contrast with WT MEFs, *Traf6*^−/−^ MEFs show no IL-6 induction in response to TNF even under a TLR2 pre-treating condition (Fig. 6C).

**Fig. 7:**
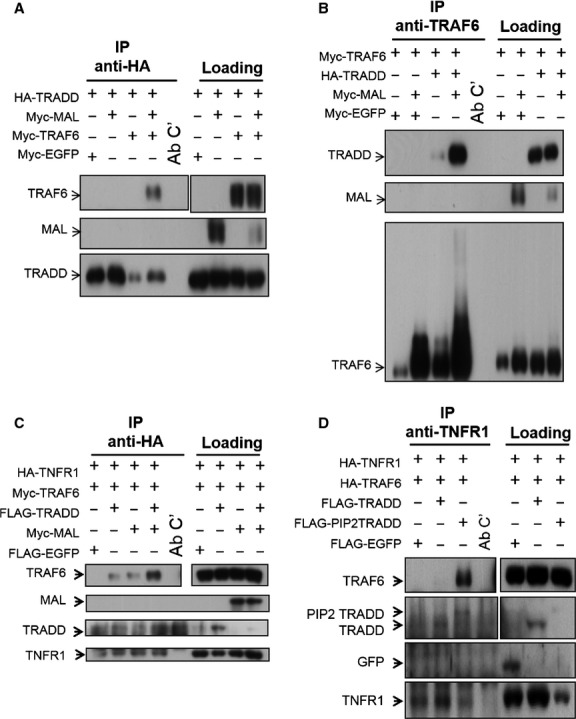
Tumour necrosis factor receptor-associated factor 6 (TRAF6) associates with TRADD, and is recruited into TNFR1 complex while MAL or PIP_2_-TRADD-GFP is co-existed in HEK293T cells. The HEK293T cells were transfected with indicative vectors for 18 hrs, cell lysates were prepared and subjected to immunoprecipitation with (**A** and **C**) anti-HA (TRADD or TNFR1 pull-down) and (**B**) anti-TRAF6 antibodies. TRADD, MAL, TRAF6 and TNFR1 were identified by Western blotting with anti-Myc or anti-HA antibodies. (**D**) HEK293T cells were transfected with indicative vectors for 18 hrs, cell lysates were prepared and subjected to immunoprecipitate with anti-TNFR1 antibody. TRADD, PIP_2_-TRADD, TRAF6 and TNFR1 were identified by Western blotting with anti-FLAG or anti-HA antibodies (Ab C’, antibody with beads control).

Our data show that TRADD is recruited proximally to membrane *via* MAL upon TLR2 stimulation. TRAF6 has been found directly interacts with MAL upon TLR2 and TLR4 stimulation [44], however, it has never been identified in TNFR1 signalsome. We next examined the relationship between TRADD, MAL and TRAF6 by immunoprecipitation analysis in HEK293T cells transfected with various combinations of effector genes. Indeed, the interaction between TRADD and TRAF6 cannot be observed in cells which only transfected with TRADD and TRAF6 vectors. Contrastingly, it was clearly detected by both directions of immunoprecipitation while a low level of MAL is simultaneously expressed in the cells (Fig. 7A and B).

To be mentioned, previously a high dose of MAL vector (1 μg) was transduced into cells to demonstrate the interaction between MAL and TRADD [29]. Here, a lower level (0.1 μg) of MAL expressing vector is used to minimize the TRADD degradation associating with MAL overexpression. Although reducing MAL level leads to the difficulty of detecting TRADD/MAL interaction, the presence of a tiny amount of MAL is sufficient to ameliorate the connection between TRADD and TRAF6.

The recruitment of TRAF6 into the TNFR1 signalsome was further examined in transfected HEK293T cells. TRAF6 is not recruited into TNFR1 signalsome in TNFR1-expressing cells co-expressed TRAF6. However, a slight recruitment of TRAF6 in TNFR1-associated complex can be observed while TRADD or MAL is co-existed. Simultaneous expressing TRADD and MAL in TRAF6/TNFR1 expressing cells leads to a dramatic increase of TRAF6 recruitment into TNFR1 signalsome (Fig. 7C). Moreover, co-expressing PIP_2_-TRADD-GFP, but not WT TRADD-GFP, is also capable of mediating the TRAF6 recruitment into TNFR1 signalsome (Fig. 7D).

**Fig. 8:**
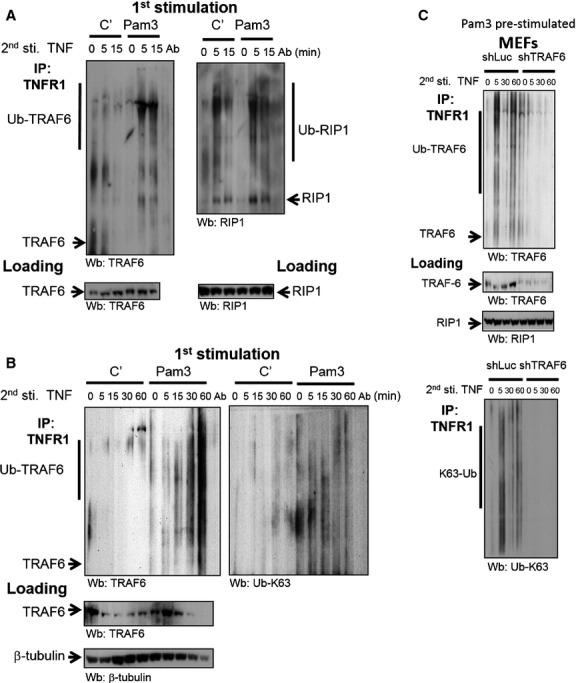
Tumour necrosis factor receptor-associated factor 6 (TRAF6) participates in TNFR1 signalsome in *Tnf*^−/−^ MEFs pre-stimulated with Pam3Csk4. *Tnf*^−/−^ MEFs were pre-stimulated with 30 ng/ml Pam3Csk4 followed by TNF treatment. Cells were harvested at indicative time-points and subjected to immunoprecipitation with anti-TNFR1 antibody. The levels of TRAF6 (**A** and **C**), RIP1 (**A**), Ub-K63 modified proteins (**B** and **C**) in the TNFR1 signalsome, and the levels of TRAF6, RIP1 (**A**–**C**) or β-tubulin (**B**) in total cell lysates were determined as loading controls by using specific antibodies.

The endogenous TRAF6 recruitment into TNFR1 signalsome was next investigated in TNF-triggered *Tnf*^−/−^ MEFs primed with or without Pam3Csk4. Quantified equal amount of proteins in whole cell lysates were used to control the initiate load of proteins for immunoprecipitation. The recruitment of TRAF6 (mostly with an ubiquitinylated profile, Ub-TRAF6) into TNFR1 complex can be observed only in Pam3Csk4 pre-treated MEFs, started at 5 min. and is sustained for at least 1 hr after TNF stimulation (Fig. 8A left and B). Contrastively, the recruitment of RIP1 and Ub-RIP1 into the TNFR1 complex is comparable between two groups (Fig. 8A right) as previously showed [29]. The ubiquitinylation changes of TNFR1-associated proteins are mostly K63-linked (Fig. 8B) but not K48-linked (data not shown). The TNFR1-associated K63-Ub modification is dramatically diminished in shTRAF6 MEFs in contrast with control shLuc cells (Fig. 8C lower), implicating that TRAF6 is a major K63-Ub-linked target recruited in the TNFR1 complex. Taken all together, membrane recruitment of TRADD mediated by MAL upon TLR2 triggering may provide a platform for TRAF6-TRADD linkage, which further conduces the consequent association of K63-Ub-linked TRAF6 into TNFR1-associated complex for signal augmentation.

## Discussion

It has been a long-time observation that TNF needs an additional signal for inducing optimal responses [23–25]. Most studies focused on investigating the molecular mechanism resulting in the cross-tolerance. We identify a route of inflammatory amplification initiated by TLR2, involving signal adaptors TRADD/MAL/TRAF6, and ends at the synergy on TNF responses. We also provide biochemical evidences which is the first time, to demonstrate the involvement of K63-linked Ub-modified TRAF6 in TNFR1 signalsome.

Multiple mechanisms may mediate the TLR-triggered synergy on TNF responses. Beyond doubt, MyD88-dependent up-regulation of endogenous TNF and TNFR would contribute to up-regulate the sequential TNF responses. Contrastingly, we identify a MyD88-independent route. In mice, serum TNF level reaches to the peak within hours in response to *in vivo* TLR stimulation [11] which perfectly echoes with the timing of newly defined TLR-mediated synergy reaching the pinnacle, suggesting that TLR signal physiologically ignites strong TNF responses within hours of infection through not only TNF auto/paracrine but also cytosolic signalling modulation. The early engagement of TRADD in TLR signalling also correspondingly matches our previous finding regarding TRADD association in TLR4 complex at 15 min. after LPS stimulation [29], implicating a novel physiological role of TRADD as an instant bridge connecting TLR and TNFR1 signals.

A TNFR to TLR signal-communication was recently revealed [32]. By contrast, our works define a crosstalk of opposite direction through the acute compartmental regulation of TRADD. Putting both models together to accomplish the crosstalk network between TLR and TNF: Initially, TLR augments TNF responses for ignition and amplification, which is sequentially followed by a TNF-GSK3-mediated signal to attenuate the NF-κB activity for termination of inflammation.

Distinct modifications with ubiquitin on cytosolic protein conduct the fates of protein from degradation to activation [45]. Recently we identified a novel tumour-suppressive role of TRADD independently of TNFR1 signalling. Intranucleus TRADD was found to modulate the interaction between p19 (Arf) and its E3 ubiquitin ligase ULF, thereby promoting p19 (Arf) protein stability and tumour suppression [40], suggesting that TRADD may play roles in modulating ubiquitinylation. TRAF6 has been recognized a crucial mediator for NF-κB activation in IL-1R and TLR signalling [43] through auto-ubiquitinylation with K63-linked polyubiquitin chains [46]. In the present study, we reveal a novel route to intensify TNF signalling through recruiting K63-Ub-linked TRAF6 into TNFR1 signalsome. The TNFR1-associated TRAF6 may serve as an additional platform for effector association and further promote TNFR downstream NF-κB and MAPK activation.

The details regarding how MAL modulates TRAF6-TRADD complex formation are largely unclear. A recent study demonstrated that prolonged MAL-mediated signalling from membrane can augment pro-inflammatory cytokine production in *p110*δ-deficient mice, perfectly echoes with our finding in PIP_2_-TRADD-GFP expressing cells [47]. We repeatedly observe a degradation of TRADD while the cells are co-expressing a high level of MAL, implicating a tight feedback exist. Accordingly, the level of PIP_2_-TRADD-GFP is consistently lower than WT TRADD-GFP expressed in all the transfection pairs we examined (Fig. 5).

Our model highlights MAL's role in linking TRADD and TRAF6, which is in conflict with previous findings by structural analyses suggested that only TRAF1 and TRAF2, but not TRAF6, could bind TRADD [48]. In fact, the direct association of TRAF6 and TRADD is hardly detectable without the help of MAL. Most of associated TRAF6 obtained in TNFR1 signalsome are heavily modified by K63-Ub, implicating the link between TRAF6 and TRADD could be indirectly through the poly-K63-Ub chains. Furthermore, TRADD has been shown interacts with TRAF3 and plays positive roles in RIG-I-like helicase-mediated antiviral activity [49]. TRAF3 is originally reported a negative regulator downstream of MyD88-mediated signalling. However, the recruitment of TRAF3 into TLR4 complex is also important for the receptorsome formation [50]. Intriguingly, both TRAF3 and TRADD are found crucial positive mediators for TRIF-mediated signalling. Whether TRAF3 is the losing piece in the synergy remains to be further investigated.

Clinically it has been a long-time observation that conditions of chronic inflammatory or autoimmune disorder patients usually go down during infection [51]. Infection-initiated flares on lupus patient's skin implicate an alert for the doctors, which could be a fatal progress pathogenically in SLE [52]. A similar phenomenon could also be observed in patients with diseases such as psoriasis, rheumatoid atherosclerosis or Crohn's disease. However, the link between infection and the degeneration of diseases remains unclear. The inflammatory amplification route we identified may illustrate the putative mechanism involved. Upon infection (TLR activation), the recruitment of TRAF6 into TNFR signalsome may play a critical role in amplifying TNF responses leading to un-controlled tissue damages.

Tumour necrosis factor antagonists are widely used to treat patients with chronic inflammatory diseases [53–55]. Depleting TNF/TNFR1 function effectively prevents the overwhelming of septic inflammation in mice. However, clinical therapies for sepsis directly targeting TNF signal showed bad prognosis and low efficiency [56,57]. In fact, TNF signal not only contributes to the pro-inflammation but also controls the termination of the prolonged and excessive inflammation. Complete down-regulation of TNF functions *via* TNF antagonists could induce a lot of side effects. The ideal treatment which can specifically block the ‘dark side’ of TNF induced by pathogens but keep the ‘bright-side’ physiological TNF functions remains to be developed. Our finding may provide an innovative approach to overcome the obstacle. Directly target the cross-talk between TLR and TNFR may inhibit the inflammatory amplification but remain other vital functions of TNF, suggesting a fascinating target for designing new therapeutics to achieve immune balance in infectious and inflammatory diseases.
